# *Leishmania aethiopica* Field Isolates Bearing an Endosymbiontic dsRNA Virus Induce Pro-inflammatory Cytokine Response

**DOI:** 10.1371/journal.pntd.0002836

**Published:** 2014-04-24

**Authors:** Haroun Zangger, Asrat Hailu, Chantal Desponds, Lon-Fye Lye, Natalia S. Akopyants, Deborah E. Dobson, Catherine Ronet, Hashim Ghalib, Stephen M. Beverley, Nicolas Fasel

**Affiliations:** 1 Department of Biochemistry, University of Lausanne, Epalinges, Vaud, Switzerland; 2 Department of Microbiology, Immunology & Parasitology, Faculty of Medicine, Addis Ababa University, Addis Ababa, Ethiopia; 3 Department of Molecular Microbiology, Washington University School of Medicine, St. Louis, Missouri, United States of America; Lancaster University, United Kingdom

## Abstract

**Background:**

Infection with *Leishmania* parasites causes mainly cutaneous lesions at the site of the sand fly bite. Inflammatory metastatic forms have been reported with *Leishmania* species such as *L. braziliensis*, *guyanensis* and *aethiopica*. Little is known about the factors underlying such exacerbated clinical presentations.

*Leishmania* RNA virus (LRV) is mainly found within South American *Leishmania braziliensis* and *guyanensis*. In a mouse model of *L. guyanensis* infection, its presence is responsible for an hyper-inflammatory response driven by the recognition of the viral dsRNA genome by the host Toll-like Receptor 3 leading to an exacerbation of the disease. In one instance, LRV was reported outside of South America, namely in the *L. major* ASKH strain from Turkmenistan, suggesting that LRV appeared before the divergence of *Leishmania* subgenera. LRV presence inside *Leishmania* parasites could be one of the factors implicated in disease severity, providing rationale for LRV screening in *L. aethiopica*.

**Methodology/Principal Findings:**

A new LRV member was identified in four *L. aethiopica* strains (LRV-*Lae*). Three LRV-*Lae* genomes were sequenced and compared to *L. guyanensis* LRV1 and *L. major* LRV2. LRV-*Lae* more closely resembled LRV2. Despite their similar genomic organization, a notable difference was observed in the region where the capsid protein and viral polymerase open reading frames overlap, with a unique −1 situation in LRV-*Lae*. *In vitro* infection of murine macrophages showed that LRV-*Lae* induced a TLR3-dependent inflammatory response as previously observed for LRV1.

**Conclusions/Significance:**

In this study, we report the presence of an immunogenic dsRNA virus in *L. aethiopica* human isolates. This is the first observation of LRV in Africa, and together with the unique description of LRV2 in Turkmenistan, it confirmed that LRV was present before the divergence of the *L. (Leishmania)* and *(Viannia)* subgenera. The potential implication of LRV-*Lae* on disease severity due to *L. aethiopica* infections is discussed.

## Author Summary

*Leishmania* RNA virus (LRV) has been detected in *Leishmania* (*Viannia*) *braziliensis* and *guyanensis* species, parasites causing not only cutaneous but also mucosal and disseminated leishmaniases. In a mouse model, the viral dsRNA genome within *L. guyanensis* parasites is recognized by host Toll-like receptor 3 (TLR3) and induces pro-inflammatory cytokines and chemokines, typically IL-6 and TNF-α, which are hallmarks of human mucosal leishmaniasis. Metastatisic complications such as mucosal and diffuse cutaneous leishmaniasis have also been described in other parts of the world, e.g. in Ethiopia. We detected LRV within *L. aethiopica* human isolates. Sequencing of three *L. aethiopica* LRVs (LRV-*Lae*) genomes confirmed that LRV-*Lae* belongs to the same *Totiviridae* family of LRVs found in South American species (LRV1) and present in a single *L. major* isolate from Turkmenistan (LRV2). LRV-*Lae* genomic organization is similar but not identical to the other LRVs, with a unique −1 frameshift situation in the overlapping region of the capsid protein/polymerase genes. Finally and similarly to *L. guyanensis* LRV1, LRV-*Lae* induced a TLR3-dependent inflammatory response in infected macrophages. The presence of LRV and its detection could be a crucial step towards better understanding the pathology spectrum of *L. aethiopica* infections.

## Introduction

In the highlands of Ethiopia, patients infected with *L. aethiopica* mostly develop localized cutaneous lesions, which can self heal. There is no accurate national figure for the overall burden of cutaneous leishmaniasis (CL) in Ethiopia, although some estimates suggest annual incidence to range from 20 to 50 thousands [1]. The prevalence varies from location to location, mostly being sporadic or endemic. In some cases, CL tends to persist and to metastasize to other parts of the body causing mucosal leishmaniasis (ML) or diffuse cutaneous leishmaniasis (DCL), two different clinical presentations. DCL starts with a cutaneous lesion that metastasizes to other cutaneous sites. Patients are anergic in response to parasite antigens and poor responders to treatment. ML patients also begin with a single lesion, but in this case infection metastasizes to mucosal tissue causing chronic inflammation and facial disfiguring lesions. The mechanisms underlying the ability of *L. aethiopica* infections to cause such pathologies are not known. The parasite genetic variability does not account for the variability in clinical presentations [2].

In South America, leishmaniases are major health problems represented by a spectrum of pathological manifestations leading to clinical forms ranging from cutaneous leishmaniasis (CL), visceral leishmaniasis (VL), mucosal (ML) or disseminated leishmaniasis (DL) depending on the infecting species [1]. ML and DL are caused primarily by infections with species of the *Leishmania* subgenus *Viannia* (e.g. *Leishmania braziliensis*, *L. panamensis* or *L. guyanensis*). One of the key questions concerning ML and DL is the basis by which some isolates are associated with metastasis in human infections. It is likely that these outcomes reflect polygenetic factors of both the human host and parasite [3]–[13]. This includes for example parasite resistance to oxidative stress [3], and a variety of host genetic susceptibilities (reviewed in [12]) related to TGF-beta signalling [6], infiltration of phagocytes [7], [11] or IL-6 production [8]. In addition, the risk of ML is increased in HIV co-infection [4] and was also found to correlate with the age, gender, nutritional status of the patients as well as duration of the disease [14]. Recently, we reported that, in *L. guyanensis* mice infection, inflammation and severity of the infection was associated with high burden of a *Leishmania* RNA virus (LRV) infecting the parasites, suggesting that LRV might also contribute to disease severity in metastasizing leishmanisis [15].

Although first described more than two decades ago [16], [17], it was only very recently reported that high burden of LRV, a member of the *Totiviridae* family, is present in parasites metastatic in hamsters and isolated from secondary lesions of ML patients [15], [18]–[20]. Thus far, these viruses have been described only in *L.* (*Viannia*) *braziliensis* and *L.* (*V.*) *guyanensis*, with the exception of one *L. major* strain from Turkmenistan (*Lmj* ASKH) [21], [22]. On the basis of sequence divergence and organization of the open reading frames, the *L.* (*Viannia*) and *L. major* viruses have been named LRV1 and LRV2 respectively. The viral particles are composed of a capsid protein (CP), an RNA-dependent RNA polymerase (RdRp) and a 5.3 kb double stranded RNA (dsRNA) genome. In most *Totiviridae*, such as in yeast, RdRp is not expressed as a single polypeptide, but as a fusion protein with CP [23]. Although the presence of a fusion protein was not directly proved for LRV *in vivo*, *in vitro* translation experiments conducted on LRV1 from *Lg* M4147 shows that the overlapping region of the two ORFs promotes translational frameshifting [24]. Another hypothesis is that an individual RdRp protein, as observed using a specific antibody, is produced after clevage of the potential fusion protein [25]. As demonstrated in the *Totiviridae* from *Helminthosporium*, RdRp could also be produced as a single protein after a termination/reinitiation mechanism [26].

In the *L. guyanensis* infection model, the dsRNA genome is recognized by the host endosomal Toll-like receptor 3 (TLR3) to induce pro-inflammatory cytokines and chemokines [15]. These TLR3-mediated immune responses render mice more susceptible to infection, and the animals develop an increased footpad swelling and parasitemia. Thus, LRV1 in *L. guyanensis* parasites subverts the host immune response to *Leishmania* and promotes parasite persistence.

In this study, we hypothesized that, because of the severity of the clinical presentations observed in *L. aethiopica* infections, a *Leishmania* RNA virus (LRV) could be present in some *L. aethiopica* strains. We showed that a virus related to *L. major* LRV2 was widespread and can evoke cytokine responses similar to those seen previously with LRV1 from *L.* (*Viannia*) [15]. This sets the stage for future studies looking at the role of LRV2 in the severity and nature of human leishmaniasis.

## Methods

### Parasite strains and cultures

Two *L. guyanensis* clones, designated here as *Lg* M4147 LRV1+ and *Lg* M4147 LRV1−, and which were previously shown to be highly- and non-infected by LRV respectively (designated as LRV^high^ and LRV^neg^ in [27]), were used as reference parasites.

Eight strains of *L. aethiopica* parasites were freshly isolated from infected patients who contracted leishmaniasis in Ethiopia (Table 1, fresh isolates). In addition, three *L. aethiopica* lines from *Leishmania* species reference centers were also used (Table 1, cryobank lines), and kindly provided by Charles Jaffe and Lee Schnur (Jerusalem, Israel).

**Table 1:** *Leishmania aethiopica* lines used in this work.

	Abbreviation	Complete code	LRV status	Pathology
**Fresh isolates**	*Lae* 077	MHOM/ET/2010/LDS077	**−**	DCL
	*Lae* 215	MHOM/ET/2011/LDS215	**−**	CL
	***Lae*** ** 303**	MHOM/ET/2011/LDS303	**+**	CL
	*Lae* 315	MHOM/ET/2011/LDS315	(**+**)	CL
	***Lae*** ** 316**	MHOM/ET/2011/LDS316	**+**	CL
	***Lae*** ** 327**	MHOM/ET/2011/LDS327	**+**	CL
	*Lae* 332	MHOM/ET/2011/LDS332	**−**	DCL
	***Lae*** ** 372**	MHOM/ET/2008/LDS372	**−**	CL
**Cryobank lines**	*Lae* L147	MHOM/ET/1972/L100	**−**	DCL
	***Lae*** ** L494**	MHOM/ET/1985/LRC-L494	**+**	CL
	*Lae* L495	MHOM/ET/1985/LRC-L495	**−**	CL

LRV status was determined by a dot blot assay (dsRNA detection) for the eight fresh isolates, and by PCR using universal LRV-specific primers on cDNA obtained from the three cryobank lines (see Methods). dsRNA was weakly detected in the *Lae* 315 strain (+). The four isolates selected for further analysis are highlighted in bold, as well as the L494 strain that was used for LRV sequencing. Parasites were isolated from patients suffering from cutaneous (CL) or diffuse cutaneous leishmaniasis (DCL) as indicated in the “pathology” column.

All parasite strains were cultivated as promastigotes at 26°C in Schneider's insect medium (Sigma) supplemented with fetal bovine serum, Hepes, penicillin/streptomycin, biopterin and hemin as described before [28].

### Detection of dsRNA by dot blot and immunofluorescence microscopy

Viral dsRNA genome was detected using the J2 monoclonal mouse antibody (English & Scientific Consulting) as described before [28]. Briefly, approximately 5×10^5^ stationary phase promastigotes (2 µg of total proteins by BCA quantification) were directly spotted on a nitrocellulose membrane for dot blot analysis. After blocking with milk, the membrane was incubated with the J2 antibody (1∶1000) that was finally recognized by an anti-mouse IgG antibody coupled to peroxidase (Promega).

For immunofluorescence microscopy (IFM), stationary phase promastigotes were fixed in formaldehyde before being attached to poly-lysine coated slides. After permeabilization and blocking steps, slides were incubated with the J2 antibody (1∶800), which was then visualized using a goat anti-mouse IgG coupled to AlexaFluor 488 (1∶600, Invitrogen).

### Viral dsRNA extraction from nucleic acids

Total nucleic acids from stationary phase promastigotes were obtained either by standard Trizol (Invitrogen) protocol or after lysis in sarkosyl followed by proteinase K and ssRNase incubation as described before [28]. After phenol-chloroform extraction and precipitation of total nucleic acids (containing genomic parasitic DNA and LRV dsRNA), parasite DNA was eliminated by a RQ-DNase treatment (Promega or Invitrogen) and LRV dsRNA was visualized on 0.8% agarose gel by staining with ethidium bromide, and purified from the gel for further cDNA preparation (below). To decrease intensity of ethidium bromide stained rRNA, and thus better visualize LRV dsRNA from the *L. aethiopica* L494 strain, total cellular RNA (after Trizol extraction) was treated with two volumes of FFS buffer (7.5M formaldehyde, 20% formamide, 0.2M sodium chloride, 30% glycerol and 0.02% bromphenol blue) at 37°C for 15 min.

### Viral genomes sequencing and comparison

The LRV-*Lae* L494 sequence was obtained by combination of total small RNA sequencing and specific-primer sequencing from cDNA. A library of small RNAs (<42 nt) was generated from total RNA (purified with Trizol, described above) using the method described by Atayde and co-workers [29]. Recent genome sequence data of the *Lae* L147 line reveals an absence of genes required for RNAi, consistent with the evolutionary position of *L. aethiopica* within the *Leishmania* clade shown previously to lack RNAi [27], and thus the small RNAs represent primarily degradation products (unpublished data), as seen previously in similar studies in *Leishmania tarentolae* which also lacks RNAi [27], [30]. The library was sequenced using Illumina HiSeq2000 technology, yielding 35.9 million reads. The 5′ and 3′ adapters were trimmed from the data and then mapped to the sequence of the *Lae* L494 PCR products described above and/or the *L. major* LRV2. From this analysis, three large contigs were obtained, and the remaining regions of the LRV2 were obtained by PCR amplification across the gaps and sequencing. This strategy allowed us to get the complete 5193 bp LRV-*Lae* L494 genome sequence (GenBank accession number: KF757256).

After purification of LRV genomic dsRNA from the infected *L. aethiopica* 303 and 327 strains (see previous section), it was quantified and used for cDNA synthesis as described before [28]. Different overlapping PCR fragments were progressively amplified from the viral cDNA and further sequenced (by Fasteris, Switzerland) to finally obtain most of the viral genomic sequence (5048 bp), including the complete open reading frames encoding the capsid protein (CP) and the RdRp (GenBank accession numbers: KF256264 and KF256265). PCR was performed as previously described [28] with an annealing temperature adapted to each set of oligonucleotides that were used (generally about 2°C below the lowest melting temperature of both primers used). All primer sets (Microsynth, Switzerland) that were used for LRV sequencing, detection and cDNA are listed in Table S1.

### Macrophage *in vitro* infection and cytokine production analysis by ELISA

Bone marrow derived macrophages (BMM) were obtained from C57BL/6 and TLR3 knock-out mice, and infected by *Leishmania* promastigotes as described before [15]. Culture supernatants were collected 24 h post-infection and analyzed for IL-6 and TNF-α cytokine production. For this purpose, ELISA kits were purchased from eBioscience and read on a Biotek Synergy HT spectrophotometer. Cytokine production was quantified relatively to purified mouse IL-6 and TNF-α standards. The number of parasites per macrophage was counted after fixation with formalhehyde followed by DAPI staining (as described in [28]). Four different pictures from each experiment were used for counting (at least 90 macrophages).

## Results

### Detection of LRV in *L. aethiopica* human fresh isolates

We surveyed eight strains of *L. aethiopica* freshly isolated from infected patients who contracted leishmaniasis in Ethiopia, six exhibiting typical CL and two with typical DCL pathologies (Table 1, fresh isolates). LRV presence was first assessed using a dot blot technique based on a monoclonal antibody (J2), which recognizes specifically dsRNA irrespective of the nucleic acid sequence [31], [32]. Here, LRV can be easily detected in minute quantities of whole parasites or from lesion biopsies (e.g. less than 100 parasites or 100 ng RNA extract in highly infected strains) [28]. Briefly, the eight freshly isolated *L. aethiopica* (*Lae*) parasites were cultured and then spotted on a nitrocellulose membrane followed by an immunoblotting assay using the J2 antibody. As positive and negative controls, we used two clonal lines shown previously to bear LRV1 (*Lg*M4147 LRV1+) or selected for loss of LRV1 (*Lg*M4147 LRV1−) [27], [28].

**Figure 1:**
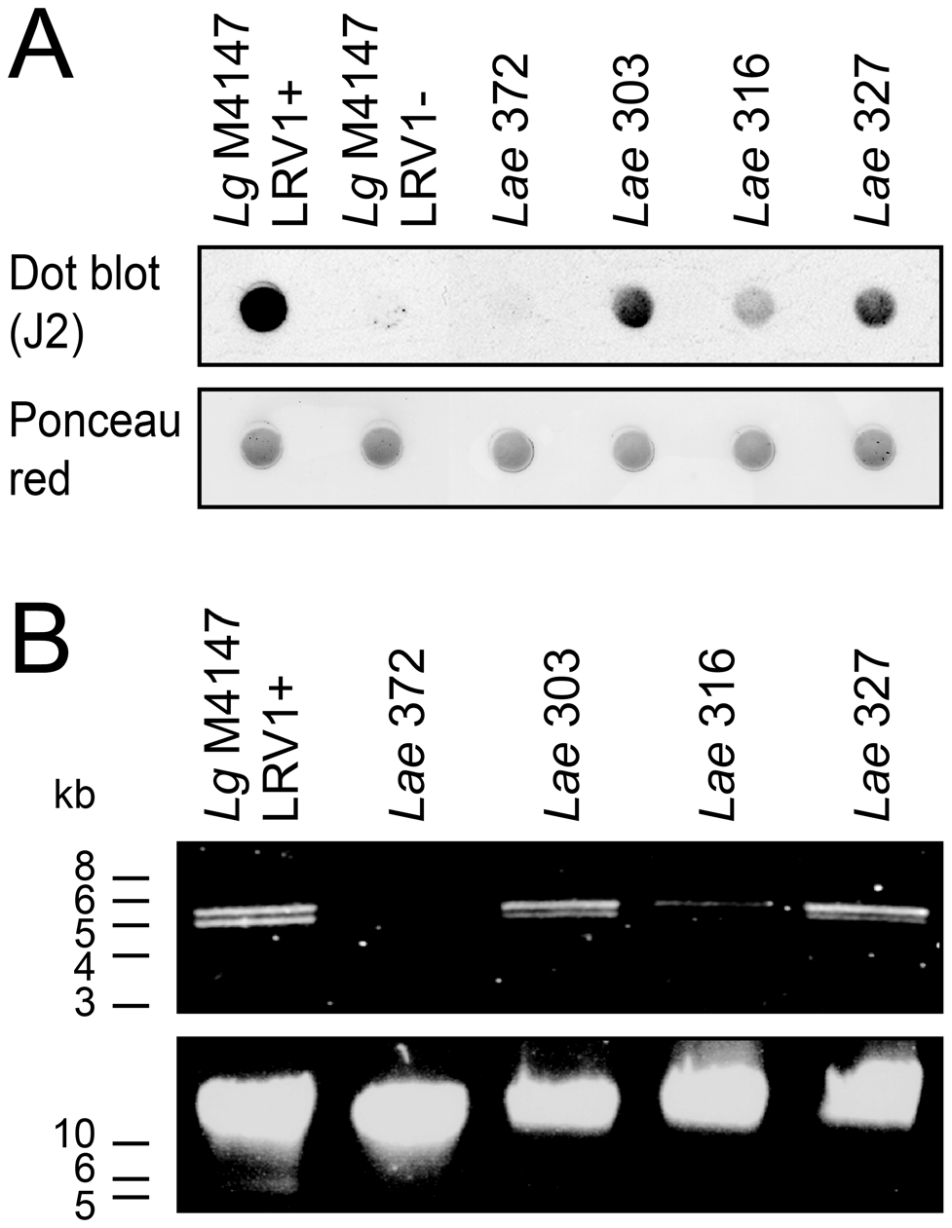
LRV is found in *L. aethiopica* field isolates. **A. LRV detection by dot blot using an anti-dsRNA antibody (J2).** Promastigotes were directly spotted onto a nitrocellulose membrane (2 µg of total protein/spot) before dsRNA detection by J2. A ponceau red staining of the membrane before J2 addition is added to demonstrate that similar amounts of parasites were loaded. **B. Viral genomic dsRNA visualization from nucleic acid extracts.** Total promastigote nucleic acids (5 µg for *Lg* and 20 µg for *Lae*) were digested with ssRNase and DNase I then migrated in a 0.8% agarose gel (upper panel). An undigested control of each sample acts as a loading control (lower panel).

Out of the eight *L. aethiopica* fresh isolates, four strains showed a detectable level of dsRNA, while all others were found negative (Table 1). The three strains that showed the strongest dsRNA reactivity (*Lae* 303, 316 and 327) as well as one negative strain (*Lae* 372) were selected for further analysis. Figure 1A shows the dsRNA detection by dot blot using the J2 antibody of these four *L. aethiopica* strains, in comparison to *Lg* M4147 reference clones. We concluded that *Lae* 303 and 327 had a higher level of dsRNA than *Lae* 316 (although weaker than the *Lg* positive control), while it was undetectable in *Lae* 372 similarly to the *Lg* negative control.

In order to demonstrate that the dsRNA detected by dot blot in these three *L. aethiopica* strains was accompanied by the presence of a dsRNA viral genome, nucleic acids were extracted and treated with DNase and ssRNase, allowing the visualization of viral dsRNA on agarose gels. As expected in the case of *Leishmania* RNA virus (LRV) infections, dsRNA molecules of approximately 5.3 kb were identified in LRV-positive *Lae* strains, only in those lines positive in anti-dsRNA tests (Figure 1B). As previously observed for other infected *L. guyanensis* and *L. braziliensis* strains [28], the LRV genome sometimes appears as a doublet in non-denaturing high-resolution gels such as that presented here. Considering the homogeneity between the viral sequences that we obtained so far (including the ones presented below for *L. aethiopica*), the doublet is unlikely to represent a mixed population of LRV. Instead, we propose that this migration pattern is due to differences in their secondary structures. Currently, it remains an open question, which deserves further analysis.

**Figure 2:**
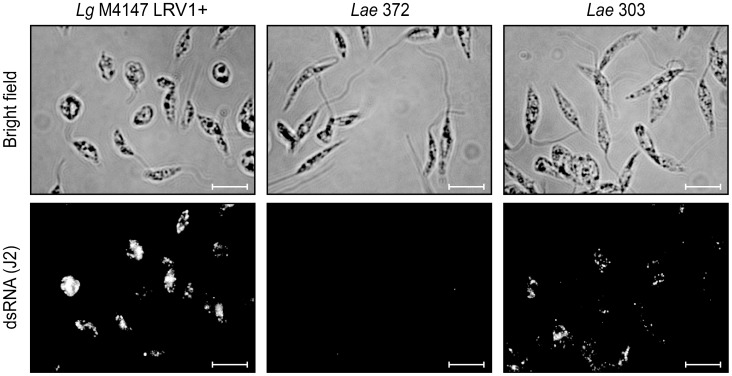
LRV-*Lae* dsRNA localization by immunofluorescence microscopy. Promastigotes were fixed with formaldehyde and spread on poly-lysine coated slides before visualization of viral dsRNA with the J2 antibody (standardized exposure time in all images: 200 ms). Scale bars: 10 µm.

The *Lae* 303 and 327 parasites harboring high dsRNA levels, as well as the LRV-negative *Lae* 372 and the *Lg* controls, were subjected to immunofluorescence microscopy to study LRV localization. As shown in Figure 2, dsRNA was detected as clusters throughout most of the cytosol in *Lae* 303 (as well as in *Lae* 327, data not shown), similar to what was seen previously with the *Lg* M4147 LRV1+ control [28]. In agreement with the prior results, dsRNA reactivity was weaker in *Lae* relative to *Lg* M4147 LRV1+ (Figure 1A), and no reactivity was visible in the LRV-negative *Lae* 372 promastigotes.

### LRV detection in a catalogued *L. aethiopica* strain

**Figure 3:**
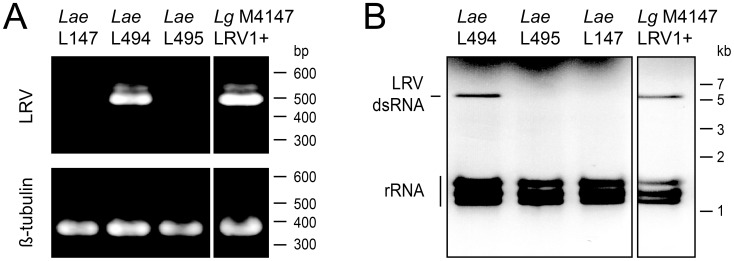
LRV detection in the *L. aethiopica* L494 strain. In addition to the three *L. aethiopica* cryobank lines tested, *Lg* M4147 LRV1+ was added as a positive control. **A. PCR amplification.** A portion of the LRV capsid protein open reading frame (489 and 486 bp for LRV1 and LRV2 respectively) was amplified from total cDNA using LRV universal primers (Table S1). As a cDNA quality control, a 372 bp fragment of the beta-tubulin gene was also amplified. **B. LRV dsRNA visualization.** Total RNA was analyzed on agarose gel. Ribosomal RNA (rRNA) and the complete 5.3 kb LRV genomic dsRNA are indicated.

Although never described in this *Leishmania* species neither on the African continent before, LRV was strikingly present in half of the recently isolated parasite strains tested. We therefore wondered if it was a particularity of this sampling, the region where samples were isolated or a general phenomenon. To this purpose, we tested already described *L. aethiopica* lines from reference centers (Table 1, cryobank lines). Three strains, isolated more than twenty years ago, were screened for LRV presence by PCR using primers specific for regions conserved across known LRV1 and LRV2 genomes (see Methods and Table S1); one of these is a WHO reference line (*Lae* L147). A specific fragment was amplified from total cDNA from one of these strains, namely *Lae* L494 (Figure 3A and Table 1), and correspondingly, this strain alone exhibited a 5.3 kb dsRNA LRV genome (Figure 3B). Thus the presence of LRVs was not uncommon in *L. aethiopica*.

### LRV-*Lae* sequence comparison to LRV1 and LRV2

**Figure 4:**
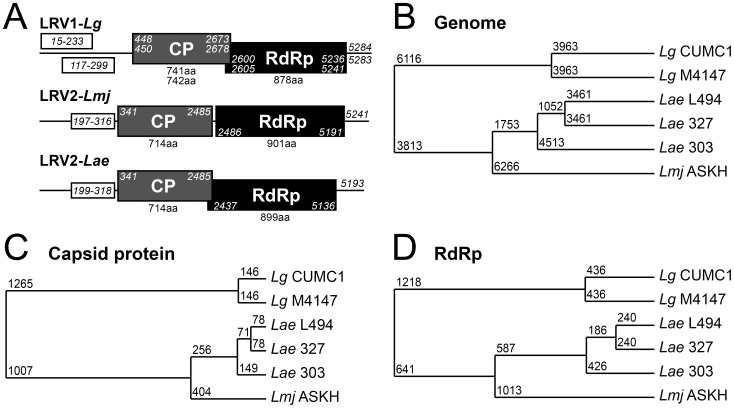
LRV2-*Lae* genome comparison to previously sequenced LRVs. **A. Comparison of LRV1-*Lg*, LRV2-*Lmj* and LRV2-*Lae* open reading frames (ORFs).** The two major ORFs encoding the capsid protein (CP) and the RNA-dependent RNA polymerase (RdRp) are represented by gray and black boxes respectively. The short ORFs upstream of the CP genes are represented by open boxes with nucleotide positions indicated in italics. The two short ORFs (15–233) and (117–299) upstream of LRV1-*Lg* capsid are deduced from LRV1-*Lg* CUMC1 and LRV1-*Lg* M4147 viral genomes respectively. **B–D. Phylogenetic analysis of** ***L. aethiopica*** **LRVs and previously sequenced LRV genomes.** Total RNA genome sequence (**B**), and the deduced capsid (**C**) and RdRp amino acid sequences (**D**) of LRVs isolated from the indicated strains were analyzed by Jalview. The average distances (using BLOSUM62) are indicated on the trees.

We determined the dsRNA sequence of the virus infecting *L. aethiopica* L494 by a combination of random small and specific primer-based RNA (cDNA) sequencing (see Methods and Table S1). This yielded a complete 5193 nucleotide genome (GenBank accession number: KF757256), designated LRV2-*Lae* L494 by the revised taxonomy proposed for LRVs as discussed below. This primary sequence was then used to design primers for sequencing most of the dsRNA genome from cDNA of two additional LRV-*Lae* isolated from *Lae* 303 and 327 (5048 bp), including the complete open reading frames for the capsid protein (CP) and the viral polymerase (RdRp) (GenBank accession numbers: KF256264 and KF256265) (Figure 4A).

The LRV-*Lae* nucleotide sequences were then aligned to those of the three complete LRV genomes in GenBank: LRV1-*Lg* CUMC1 (formerly LRV1-1), LRV1-*Lg* M4147 (formerly LRV1-4) and LRV2-*Lmj* ASKH (formerly LRV2-1) (GenBank accession numbers NC002063, NC003601 and NC002064 respectively). The overall nucleotide sequence identity amongst the three *L. aethiopica* LRVs ranged from 77 to 85%, while it was 68% with *L. major* LRV2 and 52–58% with the two *L. guyanensis* LRV1s (Table 2, genome column). Thus *L. aethiopica* LRVs were most closely related to *L. major* LRV2 at the overall nucleotide and, even more at the amino acid level as discussed below. This close relationship of LRV-*Lae* with LRV2-*Lmj* was clearly illustrated by a phylogenetic analysis (Figure 4B). Significantly, it mirrored the evolutionary relationship of *Lae* and *Lmj* parasites [33], consistent with the prevailing view that most members of the *Totiviridae* are thought to be inherited vertically and that *Leishmania* RNA viruses show relationships similar to their host [22]. For these and reasons evident below, we have assigned the *L. aethiopica* LRVs as new members of the LRV2 species of *Leishmania* RNA virus (LRV2-*Lae*).

**Table 2:** *L. aethiopica* LRV genomes analysis in comparison to LRV1 and LRV2.

			Genome	CP	RdRp
**LRV-** ***Lae***	327	303	78.0	94.5	81.4
	L494	327	85.1	99.2	89.7
	303	L494	77.2	94.1	80.8
Average			**80%**	**96%**	**84%**
**LRV2**	*Lmj* ASKH	303	68.3	79.6	61.2
		327	67.5	80.2	60.6
		L494	67.9	80.3	60.1
Average			**68%**	**80%**	**61%**
**LRV1**	*Lg* CUMC1	303	52.4	37.4	40.4
		327	52.0	36.8	39.6
		L494	51.8	36.8	40.1
	*Lg* M4147	303	51.9	35.8	40.7
		327	51.8	35.6	39.0
		L494	58.1	35.8	39.6
Average			**53%**	**36%**	**40%**

The three *L. aethiopica* LRV genome sequences (303, 327 and L494) were aligned and compared to each other (LRV-*Lae*), as well as to LRV2 from *L. major* ASKH and LRV1 from *L. guyanensis* CUMC1 and M4147 using LALIGN software from Expasy. Total nucleotide (Genome column) and deduced amino acid sequences of the capsid protein (CP column) and RNA-dependent RNA polymerase (RdRp column) were analyzed. The percentage of identical residues is indicated. Average percentage of identity for each LRV group was also included and highlighted in bold (‘average’ lines).

Conceptual translation of the LRV2-*Lae* genomes revealed the presence of two long and overlapping open reading frames (ORFs) coding for a capsid protein (CP) and an RNA-dependent RNA polymerase (RdRp) similarly to previously described LRVs (Figure 4A). The position of these ORFs in the viral genome and the size of the encoded proteins are strikingly similar to what was observed in LRV2-*Lmj*, if we admit that LRV capsid starts at an internal AUG (position 341, Figure 4A) and not at an upstream in-frame AUG, which is present in two of the LRV2-*Lae* but not in LRV2-*Lmj* (position 248). Analysis of LRV2-*Lae*303 sequence further supported our hypothesis that the AUG at nucleotide 248 is unlikely to be used, since it is followed by an in-frame stop codon upstream of the AUG at nucleotide 341 (GenBank KF256264). Therefore, in the discussion below, we took this shorter predicted protein as the LRV2-*Lae* CP.

Similarity between the LRV2 genomes also applied to an additional short ORF located upstream of the CP gene, that potentially encoded a 39 amino acid peptide highly conserved in both LRV2-*Lae* and LRV2-*Lmj* (85% identity). In contrast, the upstream short ORFs that were described in LRV1 genomes were not conserved (Figure 4A). Whether such ORFs are translated into protein is still unknown.

The amino acid sequences of the CP and RdRp from the three LRV2-*Lae* were then compared to the three available LRVs. As expected, and even more strikingly than the genome analysis, both LRV proteins from the *L. aethiopica* strains were clearly more homologous to their counterpart in *L. major* (sharing 80% and 60–61% identical residues for CP and RdRp respectively), than to the South American LRV1s (with only 36–41% of the residues being conserved for both proteins) (Table 2, CP and RdRp columns). These genome analysis also revealed that RdRp showed more diversity than CP, even between closely related strains such as the three LRV2-*Lae*, with the exception of certain highly conserved central domains (Figures S1 and S2). These include six regions that were previously reported to be conserved among various *Totiviridae*, three of them having been directly shown as critical for polymerase activity from the *S. cerevisiae* L-A totivirus [34]–[36] (Figure S3). From the CP and RdRp alignments, phylogenetic trees were constructed, again clearly dividing the LRVs into two separated groups, the New and Old World species accordingly to their parasite hosts (Figure 4C–D).

### A unique CP/RdRp frameshift in LRV2-*Lae*

**Figure 5:**
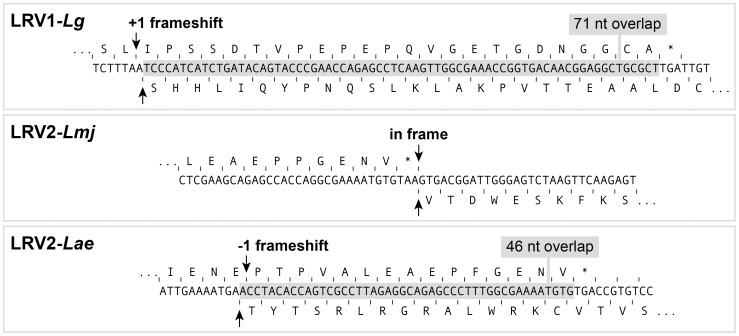
A unique LRV2-*Lae* genomic organization in the capsid protein/RdRp open reading frames switching region. The LRV genomic region coding for the end of the capsid protein (CP) and the beginning of the RdRp is shown for *L. guyanensis* M4147 (LRV1-*Lg*), *L. major* ASKH (LRV2-*Lmj*) and *L. aethiopica* 303 (LRV2-*Lae*). The corresponding amino acids of CP and RdRp are above and below the cDNA sequence respectively. CP stop codon is indicated by *. The overlapping region is shown in grey.

In LRV1-*Lg* M4147 and LRV1-*Lg* CUMC1, the open reading frames (ORFs) for CP and RdRp overlap over 71 nucleotides with a −1 frameshift (Figure 5). A similar organization was seen in LRV1s isolated from other *Viannia s*ubgenus species (manuscript in preparation). In contrast, LRV2-*Lmj* CP and RdRp are encoded by non-overlapping ORFs that are in-frame [21]. LRV2-*Lae* showed a third pattern, where the reading frames overlaped by 46 nucleotides, but now with a +1 frameshift (Figure 5). Potentially, LRVs are particularly diverse in the mechanisms used despite their close relationships. If RdRp is produced as a fusion protein with CP (as in yeast [23]), this would occur through a non-conserved mechanism of either translational frameshifting (+1 for LRV1 or −1 for LRV2-*Lae*), or via ribosomal hopping as in the case of in-frame ORFs of LRV2-*Lmj*. However it is important to note that no evidence has been provided yet establishing the existence of LRV CP-RdRp fusion proteins *in vivo* (unpublished data and [21], [24], [25]). Alternatively, RdRp could be produced as a single protein, as has been observed in *Helminthosporium* virus, which is undertaken by a termination/reinitiation mechanism [26].

### LRV2-bearing *L. aethiopica* induces TLR3-dependent cytokine induction in macrophage infections

**Figure 6:**
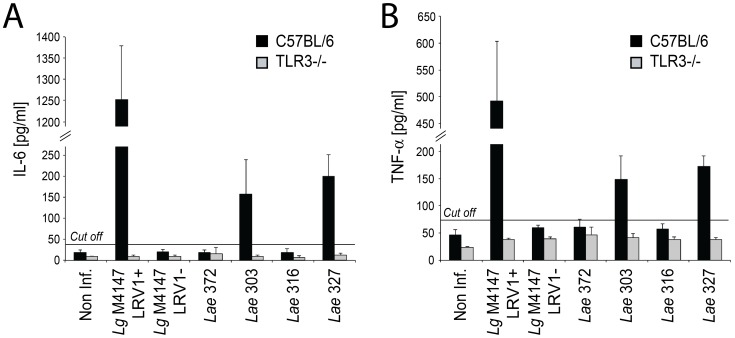
The presence of LRV2-*Lae* leads to TLR3-dependent production of pro-inflammatory cytokines by *in vitro* infected macrophages. C57BL/6 (in black) and TLR3 knock-out (in grey) murine bone marrow derived macrophages were infected by *Leishmania* promastigotes (parasite/macrophage ratio 10∶1), and the level of IL-6 (**A**) and TNF-α (**B**) in culture supernatants was measured by ELISA 24 hours post-infection. Non inf.: non-infected macrophages. The cut-off line was calculated as 3 standard deviations (SD) above the mean absorbance of the uninfected macrophage control. Average values presented were obtained from two independent experiments performed in duplicates.

Previously, we showed that LRV1-bearing *L. guyanensis* induced a TLR3-dependent hyperinflammatory response in *in vitro* infected macrophages, characterized by elevated expression of a suite of cytokines [15]. We performed similar studies here, focusing on two representative important inflammatory cytokines, IL-6 and TNF-α, by measuring their release into supernatants of *Lae*-infected macrophages. Similar to *L. guyanensis*, the infection with the two *L. aethiopica* strains harboring the highest levels of LRV (*Lae* 303 and 327) yielded significantly elevated levels of both cytokines. This was dependent on TLR3 signalling, as shown by infection of macrophages from TLR3 knock-out mice (Figure 6). Only background levels of IL-6 and TNF-α were seen with infections by the LRV-negative *Lae* 372 parasites, as it was observed with the *Lg* M4147 LRV1-negative control clone and non-infected macrophages. Interestingly, the *Lae* 316 strain, that had a low LRV load, behaved identically to a LRV-negative strain, suggesting that a minimum amount of virus was required to drive the TLR3-dependent production of IL-6 and TNF-α. The differences in cytokine levels observed with the four *Lae* strains were not due to differences in parasite uptake or survival, since a similar number of amastigotes per macrophage were quantified 24 hours post-infection with all *Lae* strains (Figure S4).

IL-6 and TNF-α production upon *Lae* 303 and 327 infection was less than that observed for *L. guyanensis*, which might be attributable to the lower LRV load in the *L. aethiopica* strains (Figures 1A and 2) and/or because of a higher parasite survival rate in the case of *L. aethiopica*. This hypothesis is supported by the observation that *L. aethiopica* survive significantly better than *L. guyanensis* parasites after macrophage infection (Figure S4). Since the activation of TLR3 by the viral dsRNA requires parasite killing and release of the virus in the phagolysosome, the different survival rates (in addition to the lower LRV load) might therefore explain the lower cytokine production observed with *L. aethiopica* LRV-infected strains in comparison to *L. guyanensis*.

*In vivo* infection experiments were also conducted in mice using these *L. aethiopica* parasites. In accordance to previous reports on such species [37]–[39], no significant footpad swelling, weight loss or any other sign of infection was measurable with any strain (data not shown). No swelling of the nose, even faint as was described in [38], was observed. This lack of clinical disease prevented the determination of the parasite load. Therefore, it was not possible to test if the presence of LRV in *L. aethiopica* and the consecutive inflammatory cytokine production had any effect in mice infection as it is observed with *L. guyanensis* parasites [15].

## Discussion

Using several approaches to detect *Leishmania* RNA virus (LRV) in whole parasite isolates [28], we showed that some strains freshly isolated from human patients infected with *L. aethiopica* contained a dsRNA virus (designated LRV-*Lae*). A complete LRV-*Lae* genome was obtained for one isolate, and more than 97% of it, including the CP-RdRp regions, for two others. These data allowed us to definitively classify the *Lae* dsRNA virus as relatives of the *Leishmaniaviru*ses LRV1 and LRV2 within the viral family *Totiviridae*, on the basis of nucleotide comparisons across the LRV genomes, the organization of the ORFs, and amino acid comparisons of the CP and RdRp proteins (Table 2, Figures 4, S1 and S2). These comparisons further showed that the *L. aethiopica* LRVs were much more closely related to *L. major* LRV2 than to the *Viannia* LRV1s, and were therefore designated as LRV2-*Lae*. In combination with the prior report of LRV2 in *L. major*, these data confirmed the presence of LRV outside of South America and the likelihood that it was present prior to the divergence of the two *L.* (*Viannia*) and *L.* (*Leishmania*) subgenera.

Despite being close to LRV2-*Lmj*, *L. aethiopica* viruses showed a striking difference in the region surrounding the CP and RdRp open reading frames. Similar to LRV1s and unlike LRV2, the reading frames from LRV2-*Lae* overlaped, but with a −1 frameshift rather than the +1 seen in all LRV1s. In contrast, the LRV2-*Lmj* CP and RdRp ORFs are non overlapping and in frame (Figure 5). Viruses are known to exhibit many forms of translational frameshifting and/or hopping [40]. Amongst *Totiviridae*, *S. cerevisiae* L-A virus and *Trichomonasvirus* TvV use frameshifting [41], [42] while others such as *Helminthosporium* virus HvV use stop/restart mechanisms [26]. LRVs are potentially diverse in the mechanisms used despite their relatively close relationships. Whether RdRp is produced as a fusion protein with CP by ribosomal frameshift/hopping or as a separate protein is still an open question that will be addressed in future studies.

Importantly, LRV2-*Lae* mirrored our previous findings with LRV1-*Lg*, and further supported the hypothesis that LRV dsRNA was a major innate immunogen as measured by the TLR3-dependent production of two key pro-inflammatory cytokines following infection of macrophages *in vitro* (Figure 6). In addition, there was likely a correlation between the viral load and the inflammatory response. It also showed for the first time that these cytokine productions were not restricted to *L. guyanensis* LRV1 but were indeed probably a general feature of LRV–infected strains.

Remarkably, LRV2-*Lae* was found in nearly half (5/11) of the *L. aethiopica* strains tested, in both ‘recent’ and archival strains (Table 1). This suggests that LRV is frequently found in *Leishmania* strains from Ethiopia, although most of the patients develop cutaneous lesions that often self-heal. As a general and primary consequence, it is unlikely that the presence of LRV2-*Lae* would by itself be sufficient to explain ML and DCL complications. Other aggravating factors may obviously combine to lead to such pathologies, as previously suggested with *L. (Viannia)* species (described in the introduction [3]–[14]). Unfortunately, our collection did not include any ML patients, therefore no conclusion could be drawn for this clinical presentation. Three samples from DCL patients were included, none of which bore LRV (or any other virus detectable by dsRNA antibody or eletrophoretic profile). While the numbers were small and as yet inconclusive, they did not point to a strong relationship between a ‘digital’ classification of human disease pathology (CL/DCL) and LRV2-*Lae* presence. A similar conclusion was reached in studies of LRV1 in South America recently [43]. This may in part reflect the difficulty of assessing human disease, as potentially CL patients may have progress to more severe disease at the time of diagnosis, parasite isolation and/or treatment. It would be of special interest to follow the CL cases from which LRV-positive parasites were isolated, and establish if the risk of ML complication is increased by the virus presence in such patients. Similarly, given the spectral nature of leishmaniasis, the range of disease severity is unlikely to be fully captured by ‘digitization’ into CL/ML. In general, a large survey of geographically and comprehensively clinically catalogued patients (CL/DCL/DL/ML) infected with LRV1 and LRV2-bearing parasites is needed to fully assess the contribution of these viruses to human pathology.

Unfortunately, animal models for *L. aethiopica* are not well developed and thus do not allow tests of the relationship between LRV and *in vivo* disease as was possible with *L. guyanensis* [15]. There, the data pointed strongly to a causal association of LRVs with disease severity and metastasis. Thus, another priority in the future is to explore and develop better models to facilitate testing of the pathogenic consequences of LRV in leishmaniasis caused by *L. aethiopica*.

## Supporting Information

Figure S1 **Capsid protein (CP) alignment of** ***L. aethiopica*** **LRVs with LRVs from** ***L. major*** **ASKH (A) and** ***L. guyanensis*** **M4147/CUMC1 (B).** Identical residues are highlighted in grey.
(JPG)

Figure S2 **RNA-dependent RNA polymerase (RdRp) alignment of** ***L. aethiopica*** **LRVs with LRVs from** ***L. major*** **ASKH (A) and** ***L. guyanensis*** **M4147/CUMC1 (B).** Identical residues are highlighted in grey.
(JPG)

Figure S3 **Conserved domains in the RNA-dependent RNA polymerase (RdRp) from LRVs and other** ***Totiviridae*** **.** RdRp sequences from *Lae* L494/*Lmj* ASKH/*Lg M4147* LRVs (indicated simply as *Lae*, *Lmj* and *Lg*) were aligned to their homologues from *S. cerevisiae* L-A virus (*Sc*) and *T. vaginalis* virus (*Tv*) using ClustalW2. The only six regions that shared at least 50% of identical residues over 5 or more consecutive amino acids are shown for each virus. Identical and similar residues are indicated by asterisks and double dots respectively. All six domains were already described as conserved among similar viral RdRp, and the third, fourth and fifth domains were directly shown to be crucial for polymerase activity [34]–[36].
(TIF)

Figure S4 ***In vitro*** **infectivity of the** ***L. aethiopica*** **strains analyzed is not affected by their LRV load after 24 hours.** Average number of parasites per bone marrow derived macrophage (BMM) were counted 24 hours post-infection from two independent experiments (same as used for cytokine measures presented in Figure 6).
(TIF)

Table S1 **Primers used for LRV-** ***Lae*** **sequencing and LRV detection by PCR.** Forward (f) and reverse (r) primer sequences were designed from the sequence of LRVs infecting the following strains (as abbreviated in the “strain” column): *L. major* ASKH, *L. guyanensis* CUMC-1/M4147 and *L. aethiopica* L494/303/327. Primer position is indicated relative to the complete *L. aethiopica* L494 LRV sequence (5193 bp). bTUBf/r primers amplify a fragment of the beta-tubulin locus of all *Leishmania* species (sequence based on *Lmj*F33.0798 gene). It was used as a quality control (QC) for cDNA preparations.
(PDF)
