# Supplementary figures and images for: Leishmania aethiopica Field Isolates Bearing an Endosymbiontic dsRNA Virus Induce Pro-inflammatory Cytokine Response
Source: PLoS Negl Trop Dis. 2014 Apr 24;8(4):e2836. doi: 10.1371/journal.pntd.0002836 (PMC3998932; doi:10.1371/journal.pntd.0002836)

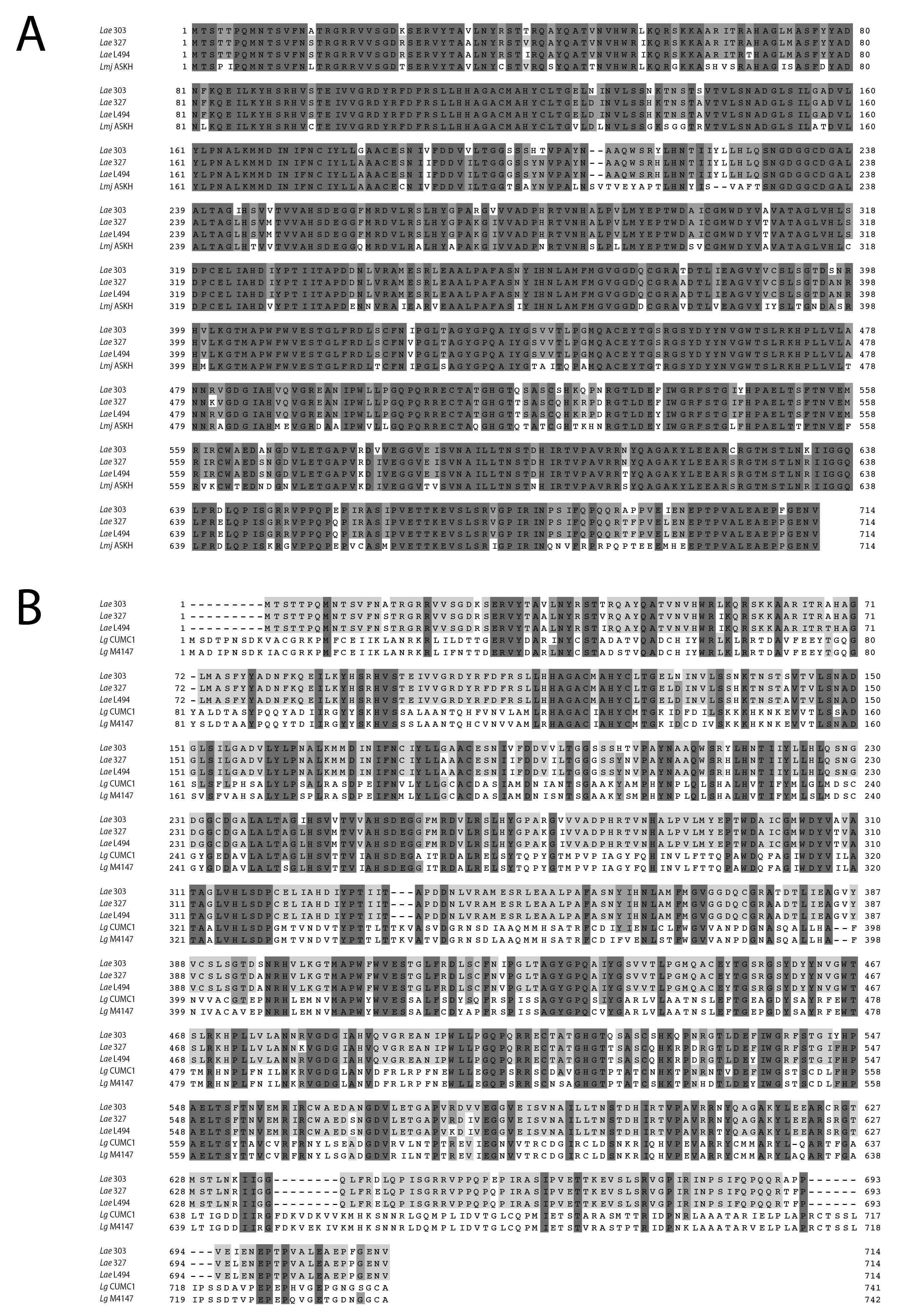

Supplement: Figure S1 — Capsid protein (CP) alignment of L. aethiopica LRVs with LRVs from L. major ASKH (A) and L. guyanensis M4147/CUMC1 (B). Identical residues are highlighted in grey. (JPG) [file pntd.0002836.s001.jpg]

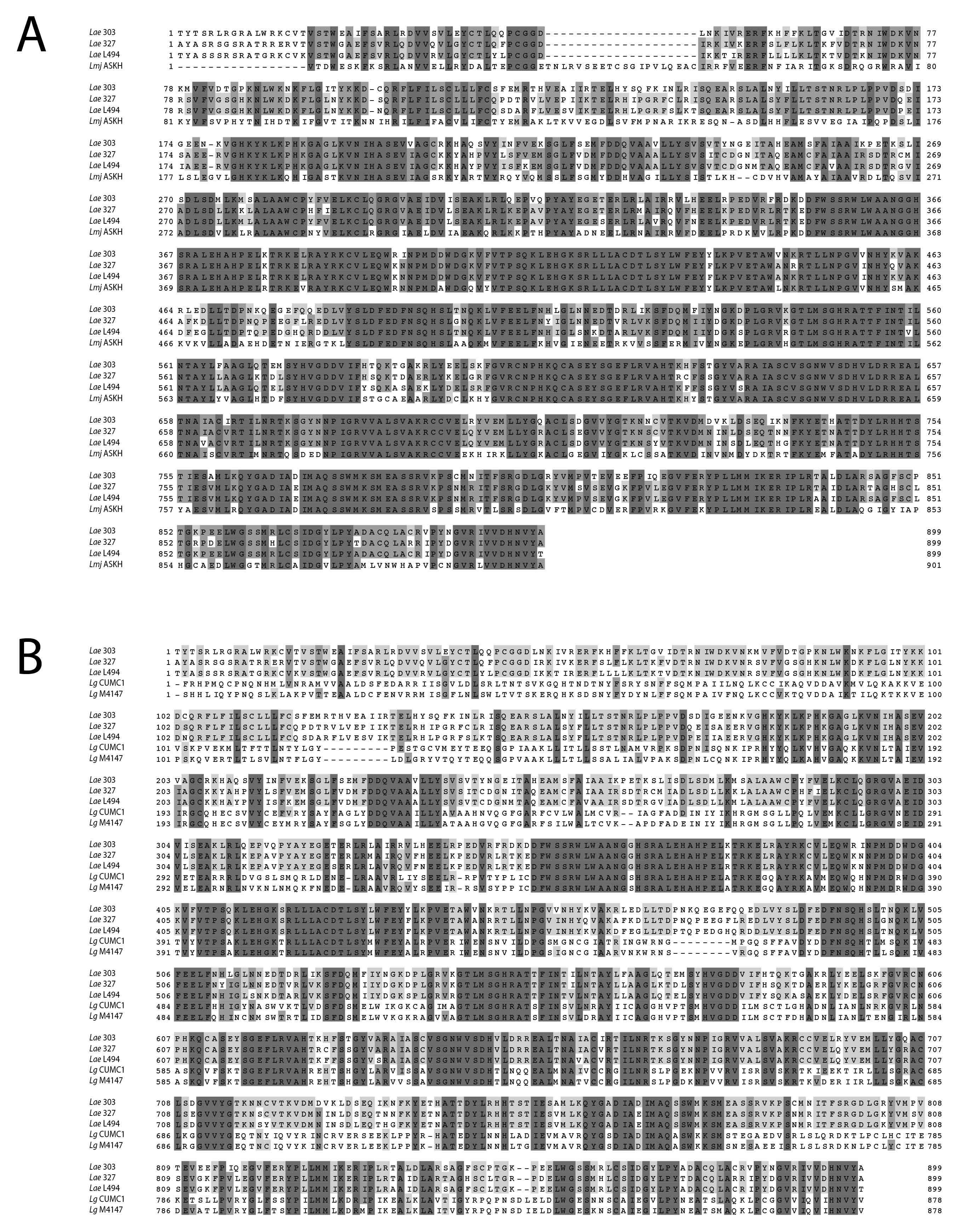

Supplement: Figure S2 — RNA-dependent RNA polymerase (RdRp) alignment of L. aethiopica LRVs with LRVs from L. major ASKH (A) and L. guyanensis M4147/CUMC1 (B). Identical residues are highlighted in grey. (JPG) [file pntd.0002836.s002.jpg]

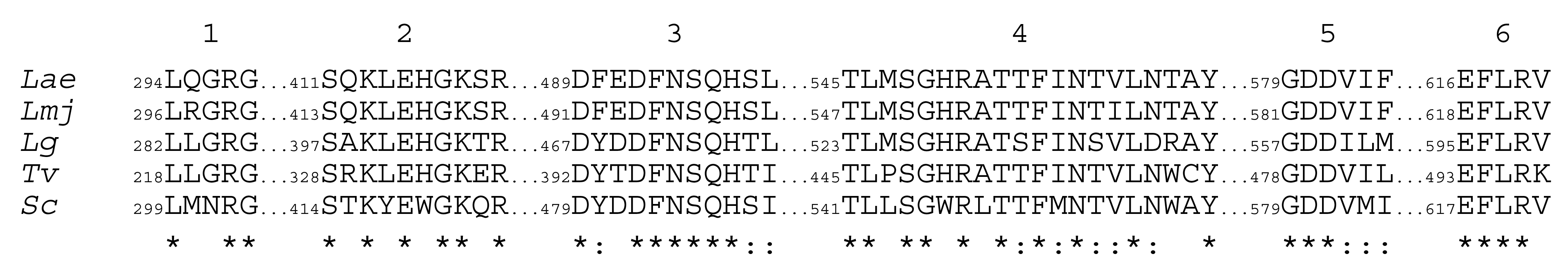

Supplement: Figure S3 — Conserved domains in the RNA-dependent RNA polymerase (RdRp) from LRVs and other Totiviridae . RdRp sequences from Lae L494/Lmj ASKH/Lg M4147 LRVs (indicated simply as Lae, Lmj and Lg) were aligned to their homologues from S. cerevisiae L-A virus (Sc) and T. vaginalis virus (Tv) using ClustalW2. The only six regions that shared at least 50% of identical residues over 5 or more consecutive amino acids are shown for each virus. Identical and similar residues are indicated by asterisks and double dots respectively. All six domains were already described as conserved among similar viral RdRp, and the third, fourth and fifth domains were directly shown to be crucial for polymerase activity [34]–[36]. (TIF) [file pntd.0002836.s003.tif]

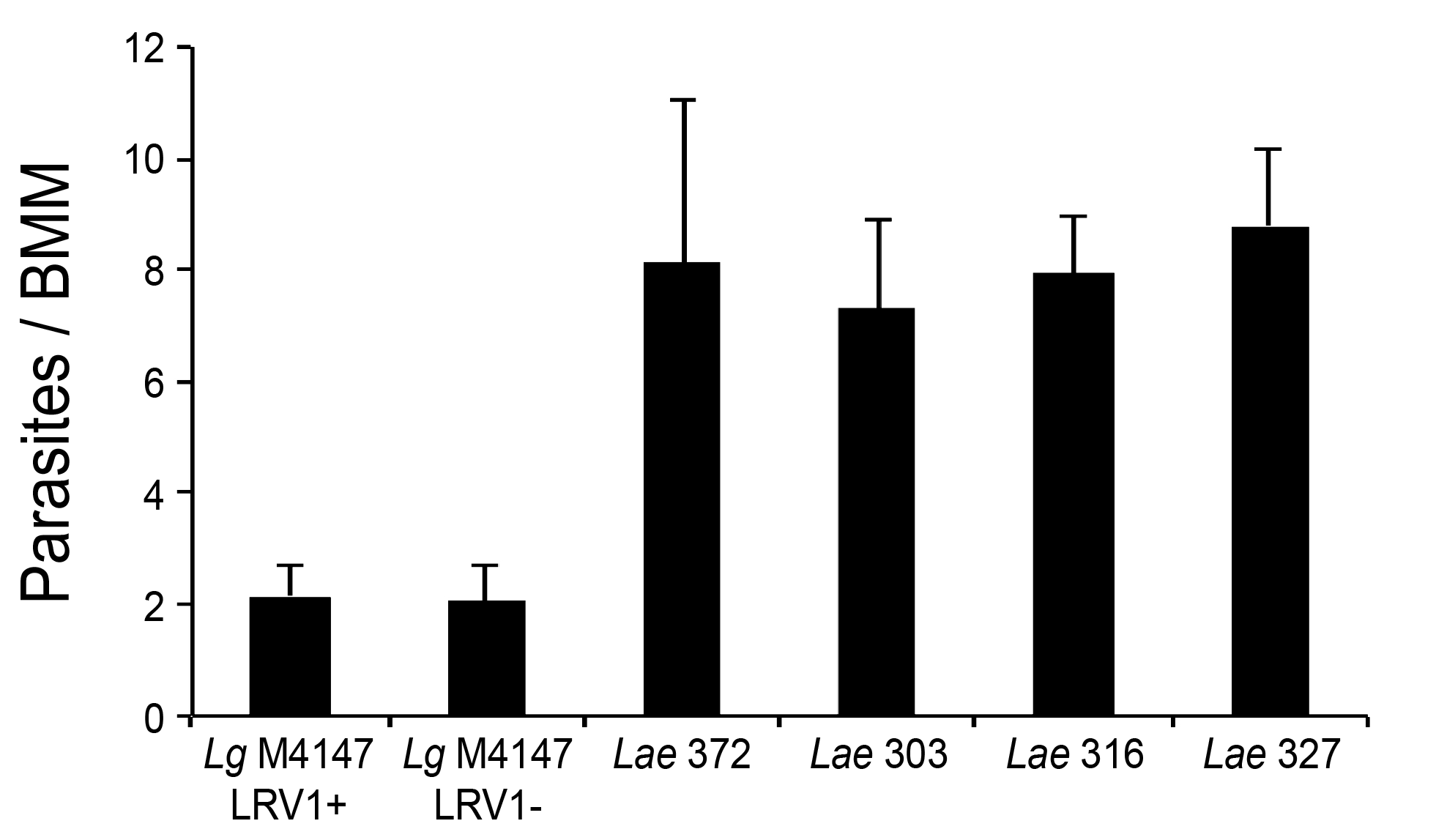

Supplement: Figure S4 — In vitro infectivity of the L. aethiopica strains analyzed is not affected by their LRV load after 24 hours. Average number of parasites per bone marrow derived macrophage (BMM) were counted 24 hours post-infection from two independent experiments (same as used for cytokine measures presented in Figure 6). (TIF) [file pntd.0002836.s004.tif]
