# Supplementary material for: Leishmania aethiopica Field Isolates Bearing an Endosymbiontic dsRNA Virus Induce Pro-inflammatory Cytokine Response
Source: PLoS Negl Trop Dis. 2014 Apr 24;8(4):e2836. doi: 10.1371/journal.pntd.0002836 (PMC3998932; doi:10.1371/journal.pntd.0002836)
Supplement: Table S1 — Primers used for LRV- Lae sequencing and LRV detection by PCR. Forward (f) and reverse (r) primer sequences were designed from the sequence of LRVs infecting the following strains (as abbreviated in the “strain” column): L. major ASKH, L. guyanensis CUMC-1/M4147 and L. aethiopica L494/303/327. Primer position is indicated relative to the complete L. aethiopica L494 LRV sequence (5193 bp). bTUBf/r primers amplify a fragment of the beta-tubulin locus of all Leishmania species (sequence based on LmjF33.0798 gene). It was used as a quality control (QC) for cDNA preparations. (PDF) [file pntd.0002836.s005.pdf]

Table S1. Primers used for LRV-*Lae* sequencing and LRV detection by PCR.

	Primer	Position	Strain	Sequence
**LRV sequencing**	1f	19-41	L494/ASKH	CGA GTG AAT CGG CCC ACT GGT AG
	2f	235-253	ASKH	CTA ACA CCT GTT GAT GCC G
	3f	341-361	L494	ATG ACC TCA ACT ACT CCC CAA
	4f	680-699	L494	CAT GCT GGT GCT TGT ATG GC
	4r	680-699	L494	GCC ATA CAA GCA CCA GCA TG
	5r	680-702	303	TGT GCC ATG CAG GCA CCG GCA TG
	6r	680-702	327	TGT GCC ATA CAA GCA CCG GCG TG
	7f	1094-1112	L494	GCC CAC AGT GAT GAA GGC G
	7r	1094-1112	L494	CGC CTT CAT CAC TGT GGG C
	8f	1935-1956	L494	TAG ATG AAT ACA TTT GGG GTA G
	8r	1935-1956	L494	CTA CCC CAA ATG TAT TCA TCT A
	9r	2566-2579	L494/303/327/ASKH	TCC CCG CCA CAG GG
	10f	2870-2885	303/327	GTC AAG AGG CAC GAT CTC
	10r	2870-2885	303/327	GAG ATC GTG CCT CTT GAC
	11f	3508-3527	ASKH	TGG CTA TGG GCG GCT AAT GG
	11r	3508-3527	ASKH	CCA TTA GCC GCC CAT AGC CA
	12r	3948-3968	303	ATT GAA TAG TTC TTC GAA GAC
	13f	4668-4688	303	CAC GAC TGA CTA TTT ACG TCA
	14r	4735-4756	ASKH	GGG CCA TGA TAT CAG CTA TGT C
	15r	5146-5169	L494	GCA CAT TAC TAG GTA CCG CCT AGC
	16r	5150-5169	ASKH	GCA CAT TGC TAG GTA CGC C
**LRV detection**	UNIVf	1089-1110	CUMC-1/M4147/ASKH	TBR TWG CRC ACA GTG AYG AAG G
	UNIVr	1553-1574	CUMC-1/M4147/ASKH	CWA CCC ARW ACC ABG GBG CCA T
**cDNA QC**	bTUBf	-	(*Lmj* Friedlin)	ACT GGA TCC ATG CGT GAG ATC GTT TCC TGC C
	bTUBr	-	(*Lmj* Friedlin)	GAC AGA TCT CAT CAA GCA CGG AGT CGA TCA GC

Forward (f) and reverse (r) primer sequences were designed from the sequence of LRVs infecting the following strains (as abbreviated in the "strain" column): *L. major* ASKH, *L. guyanensis* CUMC-1 / M4147 and *L. aethiopica* L494 / 303 / 327. Primer position is indicated relative to the complete *L. aethiopica* L494 LRV sequence (5193bp). bTUBf/r primers amplify a fragment of the beta-tubulin locus of all *Leishmania* species (sequence based on *Lmj*F33.0798 gene). It was used as a quality control (QC) for cDNA preparations.
